# Effects of blood flow restriction combined with interval training on aerobic capacity, anaerobic performance, and sport-specific performance in trained male athletes: a systematic review and meta-analysis

**DOI:** 10.3389/fphys.2026.1928418

**Published:** 2026-09-16

**Authors:** Jingyu Shen, Dan Wu, Liye Liu

**Affiliations:** 1 Department of Physical Education, Kunsan National University, Gunsan, Republic of Korea; 2 Department of Physical Education, Northeast Petroleum University, Daqing, China

**Keywords:** aerobic capacity, anaerobic performance, athletes, blood flow restriction, interval training, meta-analysis, sport-specific performance

## Abstract

This systematic review and meta-analysis evaluated the effects of blood flow restriction (BFR) combined with interval training on aerobic capacity, anaerobic performance, and sport-specific performance in trained male athletes. We searched Cochrane Library, SPORTDiscus via EBSCO, Embase, PubMed, Scopus, and Web of Science from inception to 15 May 2026. Eligible studies were randomized controlled, randomized crossover, or paired randomized trials comparing BFR plus interval-type training with interval training without BFR in trained athletes. Fifteen reports representing 13 independent studies were included. Data were synthesized using pre-post change scores. Effects were expressed as Hedges’ g standardized mean differences and pooled with random-effects models. Risk of bias was assessed with RoB 2, certainty of evidence with GRADE, and moderator effects with subgroup and sensitivity analysis. The searches identified 626 records. After removing 379 duplicates, 247 records were screened; 223 were excluded at title/abstract screening and 24 were retained for full-text assessment. BFR combined with interval training showed small statistically significant additional benefits for aerobic capacity (g = 0.30, 95% CI 0.05 to 0.55), anaerobic performance (g = 0.32, 95% CI 0.05 to 0.59), and sport-specific performance (g = 0.44, 95% CI 0.14 to 0.74). All included study-level samples were male. Statistical heterogeneity was low across primary analyses. Formal subgroup analysis did not show statistically significant subgroup differences, and sensitivity analysis did not materially change the direction of the primary findings. RoB 2 judgments were low risk for 1 study, some concerns for 11 studies, and high risk for 1 study. GRADE certainty was low for all three primary outcomes. In trained male athletes, BFR combined with interval training may provide small additional benefits for aerobic capacity, anaerobic performance, and sport-specific performance. However, the low certainty of evidence, uncertain practical significance, heterogeneous BFR prescriptions, and insufficient safety reporting do not support its immediate widespread adoption as a primary performance-enhancement strategy.

## 1 Introduction

Blood flow restriction training, originally developed in the context of KAATSU training, involves the application of an external cuff or tourniquet to the proximal portion of a limb to restrict venous return while only partially limiting arterial inflow during exercise. This manipulation changes the local circulatory environment, allowing low- or moderate-load exercise to produce a substantially greater internal physiological stimulus than the same external workload performed under unrestricted conditions (Scott et al., 2015; Patterson et al., 2019). For this reason, BFR has received considerable attention in rehabilitation and resistance-training settings, particularly when high external mechanical loads are undesirable or temporarily contraindicated (Slysz et al., 2016; Patterson et al., 2019). More recently, its potential value has also been discussed in athletic populations, where coaches and practitioners often seek methods that increase internal training stress without adding excessive musculoskeletal load (Wortman et al., 2021; Ferguson et al., 2021).

The interest in BFR is largely grounded in its capacity to disturb muscle oxygen availability and metabolic homeostasis. Under restricted blood-flow conditions, local hypoxia, metabolite accumulation, elevated perceptual stress, altered motor-unit recruitment, and cell swelling may interact with intracellular signaling pathways related to muscle protein synthesis and vascular remodeling. In resistance-based models, this environment may enhance fast-twitch fiber recruitment and anabolic signaling even when the external load is relatively low (Pearson and Hussain, 2015; Scott et al., 2015). In endurance-oriented applications, the same circulatory constraint may influence shear stress, mitochondrial signaling, and vascular adaptation, all of which are relevant to aerobic performance (Ferguson et al., 2021; Patterson et al., 2019). The interpretation becomes more complex during high-intensity intermittent exercise. Although BFR may amplify the hypoxic and metabolic stimulus of repeated efforts, excessive pressure or poorly timed cuff application can also compromise acute mechanical output, making the practical effect highly dependent on how the restriction is prescribed (Chua et al., 2022; McKee et al., 2024).

Interval training provides a particularly relevant context in which to examine this issue. Repeated bouts of high-intensity work separated by recovery periods are widely used to improve VO_2_max, power-related outcomes, and sport-specific performance. HIIT and sprint interval training are commonly implemented to target aerobic and metabolic adaptations (Laursen and Jenkins, 2002; Buchheit and Laursen, 2013a; MacInnis and Gibala, 2017), whereas repeated-sprint training and small-sided games offer more sport-specific intermittent stimuli for team-sport athletes (Girard et al., 2011; Bishop et al., 2011; Hill-Haas et al., 2011). Adding BFR to these formats is therefore appealing: in theory, it could intensify the internal hypoxic and metabolic demand of a session without requiring a proportional increase in external running, cycling, or mechanical workload. This assumption, however, should not be accepted too easily in trained athletes. Their baseline fitness, tolerance to high-intensity work, and repeated exposure to demanding training may reduce the margin for additional adaptation. The response may also vary according to cuff pressure, cuff width, inflation timing, exercise modality, training frequency, total number of sessions, and the outcome selected for evaluation. Existing studies have already applied BFR to running, cycling, repeated-sprint, and sport-specific interval models, reflecting both the practical appeal and the methodological diversity of this intervention (Taylor et al., 2016; Chen et al., 2022a; McKee et al., 2024; Liu et al., 2026).

Previous reviews generally suggest that BFR exercise can produce beneficial physiological or performance-related effects, but their conclusions are not fully aligned with the specific question addressed here. Some reviews have pooled different exercise modalities or combined athletic and non-athletic populations, which limits their direct relevance to trained performers (Slysz et al., 2016; Wortman et al., 2021). Others have focused more narrowly on BFR endurance training or BFR-HIIT and have supported the plausibility of aerobic or performance benefits, while also emphasizing substantial heterogeneity in participants, protocols, and comparator conditions (Bennett and Slattery, 2019; Castilla-Lopez et al., 2022; Chua et al., 2022). What remains unclear is whether BFR provides a measurable advantage beyond interval training alone in trained athletes, and whether this effect is moderated by practical prescription variables such as pressure setting, training frequency, total training exposure, exercise modality, or the timing of BFR application.

Therefore, the present review aimed to evaluate the effects of blood flow restriction combined with interval training on performance- and physiology-related outcomes in trained athletes, and to explore whether intervention characteristics influence the magnitude of these effects.

## 2 Methods

### 2.1 Reporting framework and protocol

This systematic review and meta-analysis was conducted and reported in accordance with the Preferred Reporting Items for Systematic Reviews and Meta-Analyses (PRISMA) 2020 statement and relevant recommendations from the Cochrane Handbook for Systematic Reviews of Interventions (Page et al., 2021; Higgins et al., 2024). The review protocol was registered in PROSPERO (CRD420261392286).

### 2.2 Search strategy

Electronic searches were performed from database inception to 15 May 2026 in PubMed, SPORTDiscus via EBSCO, Embase, Scopus, Cochrane Library, and Web of Science. The search strategy followed PRISMA and Cochrane guidance for identifying intervention studies and combined controlled vocabulary and free-text terms related to blood flow restriction and occlusion training, interval-type exercise, and athletic populations (Page et al., 2021; Higgins et al., 2024). Core search concepts included “blood flow restriction”, “BFR”, “occlusion”, “vascular occlusion”, “KAATSU”, “interval training”, “high-intensity interval training”, “HIIT”, “sprint interval training”, “SIT”, “repeated sprint”, “athlete”, “trained”, “competitive”, “elite”, and “professional”. The complete database-specific search strategies are provided in **Supplementary File 1**.

### 2.3 Eligibility criteria

Studies were eligible if they met the following PICOS criteria.

Population: trained or competitive athletes currently engaged in organized sport training and/or formal competition, described as trained, competitive, highly trained, elite, professional, or equivalent.

Intervention: blood flow restriction combined with structured interval-type training, including HIIT, SIT, repeated-sprint training, interval running, interval cycling, Tabata-type protocols, small-sided games, or comparable intermittent exercise protocols.

Comparator: the same or a closely matched interval-training protocol performed without BFR.

Outcomes: at least one performance-related outcome, including aerobic capacity, anaerobic performance, or sport-specific performance. Eligible measures included VO_2_max/VO_2_peak, maximal aerobic speed or power, Wingate-derived outcomes, sprint performance, repeated-sprint ability, time-trial performance, maximal running performance, or sport-specific field/laboratory performance tests.

Study design: randomized controlled trials, randomized crossover trials, or pair-matched randomized trials.

Studies were excluded if they: enrolled non-athlete, sedentary, recreationally active, clinical, rehabilitation, or general healthy populations; used BFR without an eligible interval-training intervention; examined only acute single-session responses without a training period; reported no eligible performance outcome; were reviews, meta-analyses, case reports, protocols, editorials, commentaries, animal studies, or cell studies; used duplicate or overlapping datasets already represented by a more complete or relevant report; or were not published in English.

### 2.4 Study selection

Database records were imported into Zotero for reference management, duplicate checking, and organization of title-and-abstract screening. After deduplication, two authors (JS and DW) independently screened titles and abstracts. Full texts were obtained for potentially eligible reports. Disagreements were resolved by discussion or by consultation with a third author (LL). Reasons for full-text exclusion were recorded.

### 2.5 Data extraction

Data extraction was conducted independently by two authors (JS and DW) using a prespecified extraction template. Any discrepancies were settled through discussion. A third author (LL) was consulted if consensus could not be reached. Numerical data were extracted only when the relevant values were explicitly reported in the article PDF, tables, or other traceable full-text materials. Values presented only in figures were not estimated using graph digitization software, and unavailable data were recorded as missing rather than inferred. The extraction template covered study design, sample size, athlete characteristics, sport, competitive level, country, interval-training format, experimental and comparator group characteristics, BFR timing, cuff position, cuff width, pressure prescription method, pressure values and units, training intensity, intervention duration, weekly frequency, total number of sessions, training protocol, outcome name, outcome category, and group-level pre- and post-intervention means, SDs, and sample sizes.

For each eligible comparison, one effect estimate was selected within each outcome domain to avoid double counting. When several outcomes from the same domain were reported, selection followed a prespecified hierarchy. Outcomes identified as primary in the original study were prioritized; when no primary outcome was stated, the outcome judged to be most directly relevant, comparable across studies, and sufficiently reported was selected. For aerobic capacity, directly measured VO_2_max or VO_2_peak was preferred over estimated VO_2_max, maximal aerobic speed, or maximal aerobic power. For anaerobic performance, Wingate-derived peak or mean power and comparable maximal power outcomes were prioritized over repeated-sprint decrement or isolated strength outcomes when more than one eligible measure was available. For sport-specific performance, time-trial, sprint, repeated-sprint, maximal running or cycling, and sport-specific endurance tests were selected according to their sport relevance, cross-study comparability, and data completeness.

The primary analysis was based on change scores because the review focused on training-induced differences between BFR and non-BFR interval-training conditions. Change scores were calculated as post-intervention values minus baseline values. For outcomes in which lower values represented better performance, the direction of the change score was reversed before effect-size calculation, ensuring that positive Hedges’ g values consistently favored BFR combined with interval training. When standard errors were reported, SDs were derived by multiplying the SE by the square root of the corresponding sample size. If change SDs were not available, they were estimated from pre- and post-intervention SDs using the recommended correlation-based formula: SD_change = sqrt(SD_pre² + SD_post² − 2 × r × SD_pre × SD_post), with r = 0.5 used in the primary analysis (Higgins et al., 2024; Borenstein et al., 2009). Sensitivity analysis repeated the meta-analysis using r = 0.3 and r = 0.7.

### 2.6 Risk of bias assessment

Risk of bias was assessed using the revised Cochrane risk-of-bias tool for randomized trials (RoB 2), targeting the effect of assignment to intervention (Sterne et al., 2019; Higgins et al., 2024). Two authors (JS and DW) independently assessed the five RoB 2 domains: bias arising from the randomization process, bias due to deviations from intended interventions, bias due to missing outcome data, bias in measurement of the outcome, and bias in selection of the reported result. Domain-level judgments were combined according to RoB 2 guidance to assign an overall judgment of low risk of bias, some concerns, or high risk of bias. Detailed study-level RoB 2 assessments and supporting judgments are provided in **Supplementary Table 1**.

### 2.7 Certainty of evidence assessment

Certainty of evidence was assessed with the Grading of Recommendations Assessment, Development and Evaluation (GRADE) approach for each primary outcome: aerobic capacity, anaerobic performance, and sport-specific performance (Guyatt et al., 2011; Higgins et al., 2024). Because the included evidence came primarily from randomized trials, the starting certainty was high and was downgraded when warranted for risk of bias, inconsistency, indirectness, imprecision, or publication bias. Final certainty ratings were categorized as high, moderate, low, or very low.

### 2.8 Data analysis

All statistical analyses were run from reproducible R scripts using R version 4.6.0. The analysis input, scripts, package versions, output tables, figures, and session information were saved to support reproducibility. The analysis dataset used for the meta-analysis is provided in **Supplementary Data Sheet 1**. Effects were expressed as Hedges’ g standardized mean differences calculated from change scores, with positive values favoring BFR plus interval training (Hedges and Olkin, 1985; Borenstein et al., 2009). Random-effects models were fitted with restricted maximum likelihood using the ‘metafor’ package (Viechtbauer, 2010; Veroniki et al., 2016). Separate primary analyses were conducted for aerobic capacity, anaerobic performance, and sport-specific performance. For each primary outcome, the analysis reported Hedges’ g, 95% CI, p value, τ², I², and Q statistic (Higgins et al., 2003).

Formal subgroup analyses were performed for pressure setting method, training frequency, and total training sessions. Pressure setting was categorized as occlusion-based pressure when based on %AOP or %LOP and non-occlusion-based pressure when based on fixed mmHg, progressive fixed mmHg, or systolic blood pressure multiples. Training frequency was categorized as ≤2 sessions/week or ≥3 sessions/week. Total training sessions were calculated from training duration and frequency and categorized as <10 sessions or ≥10 sessions. BFR timing was examined only as an exploratory subgroup analysis because the distribution of BFR-t, BFR-c, and BFR-r categories was unbalanced.

Sensitivity analysis examined three prespecified issues: exclusion of the RoB 2 high-risk study, handling of the shared control group in Giovanna et al. (2022) by retaining only BFRG or only BFRrG, and alternative assumed pre-post correlations for change SD estimation (r = 0.3 and r = 0.7). Funnel plots and Egger regression tests were performed only when an outcome included at least 10 effect estimates, because small-study effect tests have limited interpretability with fewer studies (Egger et al., 1997; Higgins et al., 2024).

## 3 Results

### 3.1 Study identification and selection

The database search identified 626 records. After 379 duplicates were removed, 247 records were screened by title and abstract. Of these, 223 records were excluded, and 24 reports were retrieved for full-text assessment. Nine reports were excluded at the full-text stage. The final review included 15 reports representing 13 independent studies. The study selection process is presented in **Figure 1**.

**Figure 1:**
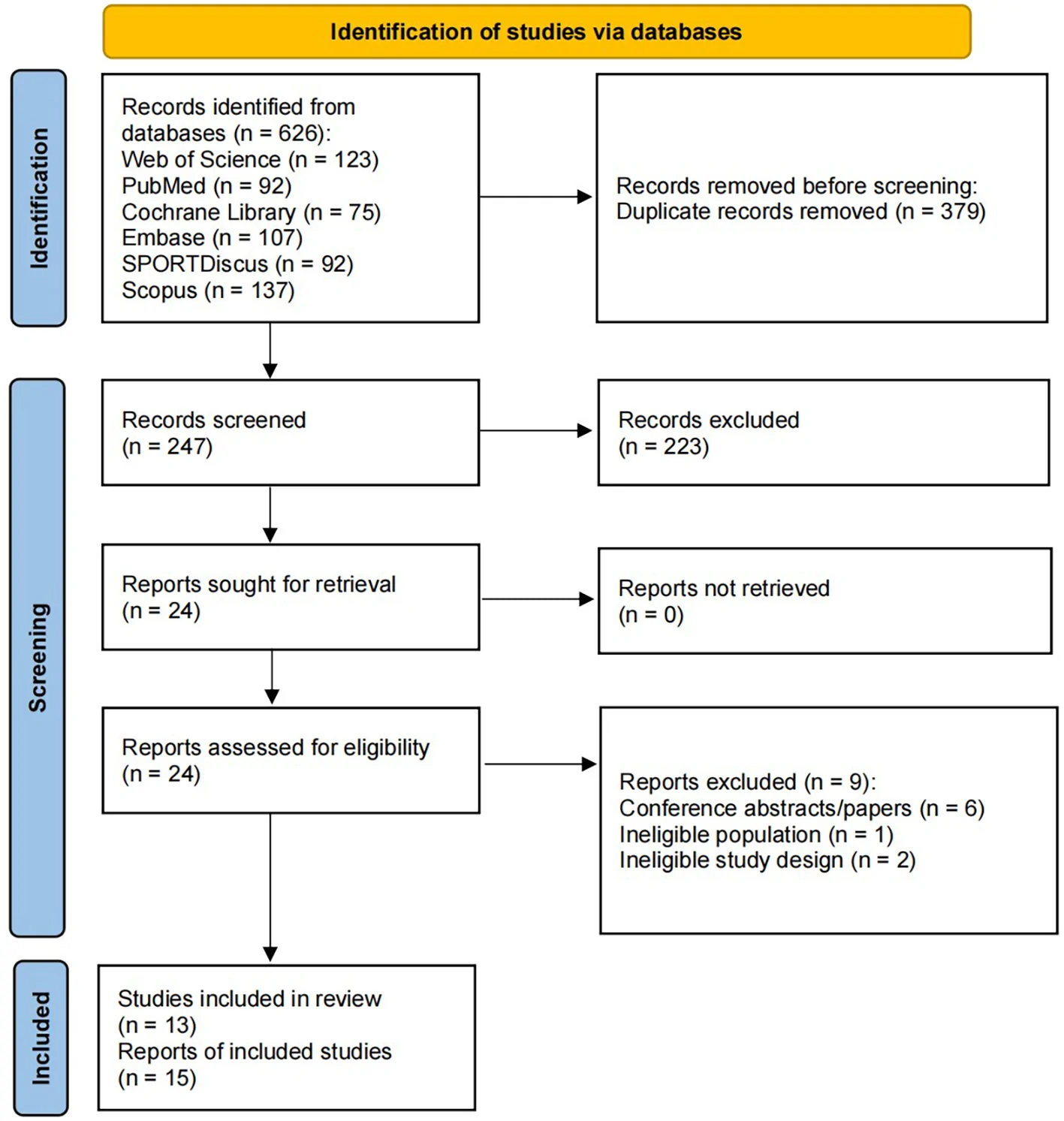
PRISMA flow diagram of study selection. Records were identified from six electronic databases, duplicates were removed, and eligible reports were screened according to PICOS criteria.

### 3.2 Characteristics of the included studies

The included reports described 347 participants across all reported trial arms. For the meta-analysis, only BFR arms and eligible non-BFR interval-training comparator arms were extracted, so comparison-level sample sizes did not always match the full-trial sample sizes, particularly in multi-arm studies. The 15 reports represented 13 independent studies: Taylor et al. (2016); Amani et al. (2018); Elgammal et al. (2020); Giovanna et al. (2022); Tangchaisuriya et al. (2022); Chen et al. (2022a); Chen et al. (2022b); McKee et al. (2024); McKee et al. (2025); Xia et al. (2025); Chen et al. (2025); Bourgeois et al. (2025); Liu et al. (2026); Castilla-Lopez et al. (2026), and Zambolin et al. (2026). Chen et al. (2022a) and Chen et al. (2022b) were treated as reports from the same independent study because participant overlap was identified. McKee et al. (2024) and McKee et al. (2025) were also treated as linked reports and were not double-counted in the primary effect estimates. Domain-specific analysis samples were smaller because not all studies contributed data to every outcome domain, and because shared-control handling affected analysis-level sample sizes.

All included study-level samples were male, and no female subgroup data were available for separate extraction. Participants were described as competitive, highly trained, well trained, semiprofessional, national-level, or provincial-level athletes. The represented sports included cycling or road cycling, endurance running, soccer or football, basketball, badminton, Australian Rules or mixed team-sport disciplines, and mixed endurance-sport samples. Intervention formats varied across studies and included sprint interval training, repeated-sprint training, HIIT, moderate-intensity interval training, Tabata training, interval running, and small-sided games. Apart from one 6-day microcycle, intervention duration ranged from 2 to 12 weeks. Training frequency ranged from 2 to 4 sessions per week, with one 6-day microcycle prescribing five sessions. Total planned training exposure ranged from 5 to 24 sessions.

BFR was applied to the lower limb, most commonly at the proximal thigh when cuff position was specified. Cuff width was not consistently reported. Where this information was available, nominal cuff widths ranged from 5 to 14.2 cm, although some studies reported cuff length or bladder dimensions rather than cuff width. Pressure prescription also varied substantially across studies. Methods included fixed mmHg pressures, progressive fixed mmHg pressures, systolic blood pressure multiples, percentage of arterial occlusion pressure (%AOP), and percentage of limb occlusion pressure (%LOP). Reported pressures ranged from 100 to 220 mmHg for fixed or progressive protocols, 30% to 70% AOP, approximately 80% LOP, 1.3 times resting systolic blood pressure, or 100% to 130% of estimated leg systolic blood pressure.

The primary meta-analyses included 30 effect estimates: 11 for aerobic capacity, 11 for anaerobic performance, and 8 for sport-specific performance. Across the main-analysis dataset, 20 distinct outcome names were extracted, including 4 aerobic-capacity measures, 8 anaerobic-performance measures, and 8 sport-specific performance measures. Detailed study and intervention characteristics are provided in **Tables 1**, **2**; full intervention protocols and BFR parameters are provided in **Supplementary Table 2**.

**Table 1:** Included study characteristics.

Study	Country	Study design	Comparison-level N	BFR group n	Control group n	Age	Sex	Sport	Competitive level
Taylor et al., 2016	United Kingdom	RCT	20	10	10	BFR 26 ± 5; con 27 ± 7 years	male	Cycling	trained men; cycling 120 ± 66 km/week
Amani et al., 2018	Iran	RCT	19	10	9	23.89 ± 2.26 years (overall)	male	Soccer/football	well-trained football players; at least 7 years football experience
Elgammal et al., 2020	Egypt	RCT	24	12	12	22.3 ± 2.4 years (overall)	male	Basketball	university basketball team; highly trained about 10 h/week; 12 years training experience
Giovanna et al., 2022	Switzerland	RCT	29	10 + 10	9	BFRG 23.9 ± 3.8; CG 30.2 ± 9.9 years	male	Cycling/endurance-trained athletes	endurance-trained athletes
Tangchaisuriya et al., 2022	Thailand	RCT	34	17	17	35–49 years	male	Road cycling	masters road cyclists
Chen et al., 2022a, b	Taiwan (China)	pair-matched randomized trial	20	10	10	RIT-BFR 21.5 ± 2.2; RIT 21.6 ± 2.1 years	male	Endurance running	endurance-trained male runners
McKee et al., 2024/2025	Australia	RCT	26	13	13	BFR 21 (5); non-BFR 20 (4) years	male	Team sports/Australian Rules	22 semiprofessional and 4 amateur adult male team-sport players
Xia et al., 2025	China	RCT	30	15	15	20.4 ± 1.2 years (overall)	male	Badminton	male collegiate badminton players; provincial level or higher competition ranking
Chen et al., 2025	China	RCT	33	17	16	BFR-MIIT 19.70 ± 1.31; HIIT 19.87 ± 1.25 years	male	Basketball	Tier 3 highly trained/national level male college basketball athletes
Bourgeois et al., 2025	Canada	RCT	18	10	8	24.9 years (SE 3.5; overall)	male	Endurance sport (cycling/running)	endurance-trained males; >2 years competitive training history
Liu et al., 2026	China	RCT	23	11	12	21.0 ± 1.6 years (overall)	male	Basketball	Tier 3 national-level; well-trained collegiate or provincial players
Castilla-Lopez et al., 2026	Spain	RCT	19	10	9	16.1 ± 0.6 years (overall)	male	Soccer	national-level male academy soccer players
Zambolin et al., 2026	Norway	RCT	17	8	9	BFR 29 ± 9; HIIT 31 ± 8 years	male	Road and mountain-bike cycling	national-level well-trained cyclists

For multi-arm studies, N refers to the eligible BFR and non-BFR comparator arms included in this review, not necessarily the full trial sample. For Giovanna et al. (2022), two BFR arms shared one control group; the control sample size was split in the primary analysis when both BFR comparisons were included.

**Table 2:** Intervention protocols and BFR parameters.

Study	Interval type	BFR timing	Duration	Frequency	Sessions	Pressure method	Pressure
Taylor et al., 2016	sprint interval training	During recovery/post-exercise	4 weeks	2 sessions/week	8	Fixed absolute pressure	130 mmHg
Amani et al., 2018	interval running	During exercise	2 weeks	4 sessions/week	8	Fixed absolute pressure	140–180 mmHg
Elgammal et al., 2020	repeated-sprint training	During exercise	4 weeks	3 sessions/week	12	Progressive fixed absolute pressure	100–160 mmHg
Giovanna et al., 2022	repeated-sprint training	During exercise	2 weeks	3 sessions/week	6	%AOP-based	45% AOP; 88.2 ± 10.1 mmHg
Giovanna et al., 2022	repeated-sprint training	During recovery/post-exercise	2 weeks	3 sessions/week	6	%AOP-based	45% AOP; 88.2 ± 10.1 mmHg
Tangchaisuriya et al., 2022	HIIT	During exercise	12 weeks	2 lab HIIT/BFR sessions/week	24	%AOP-based	30% AOP
Chen et al., 2022a, b	interval running	Continuous	8 weeks	3 sessions/week	24	SBP-based	1.3 × resting SBP; 154 ± 6 mmHg
McKee et al., 2024/2025	repeated-sprint training	Continuous	3 weeks	3 sessions/week	9	%AOP-based	45% AOP; 72 (6) mmHg
Xia et al., 2025	Tabata	During exercise	6 weeks	3 sessions/week	18	%AOP-based	60% AOP
Chen et al., 2025	HIIT/MIIT	During exercise	6 weeks	2 sessions/week	12	Fixed absolute pressure	180–200 mmHg
Bourgeois et al., 2025	HIIT	During exercise	3 weeks	3 sessions/week	9	%AOP-based	50-70% AOP
Liu et al., 2026	small-sided games	During exercise	4 weeks	2 sessions/week	8	SBP-based	100-130% estimated leg SBP
Castilla-Lopez et al., 2026	interval running	During exercise	6 weeks	2 sessions/week	12	%AOP-based	60% AOP
Zambolin et al., 2026	HIIT	During exercise	6-day microcycle	5 sessions/6 days	5	%LOP-based	180–220 mmHg; about 80% LOP

NR, not reported; AOP, arterial occlusion pressure; LOP, limb occlusion pressure; SBP, systolic blood pressure. Full intervention details, cuff position, cuff width, exercise intensity, comparator protocols, and extracted outcomes are provided in **Supplementary Table 2**.

### 3.3 Quality assessment of the included studies

The RoB 2 assessment classified one study as low risk, 11 studies as having some concerns, and one study as high risk. Risk of bias was assessed for the outcome selected from each study for the primary meta-analysis. The high-risk judgment was assigned to McKee et al. (2024), mainly because of concerns related to deviations from the intended intervention and missing or excluded outcome data after the trial had started. Across the remaining studies, the most common reasons for some concerns were insufficient reporting of allocation concealment, limited information on prespecified analysis plans, incomplete details on outcome measurement, and, in some cases, unclear reporting of deviations from the intended intervention. Because participant blinding is inherently difficult in BFR training studies, lack of blinding alone was not automatically judged as high risk. Instead, judgments were upgraded when the absence of blinding was accompanied by plausible risks arising from deviations from intended interventions, missing outcome data, or effort-dependent outcome measurement. The RoB 2 summary is presented in **Figure 2**.

**Figure 2:**
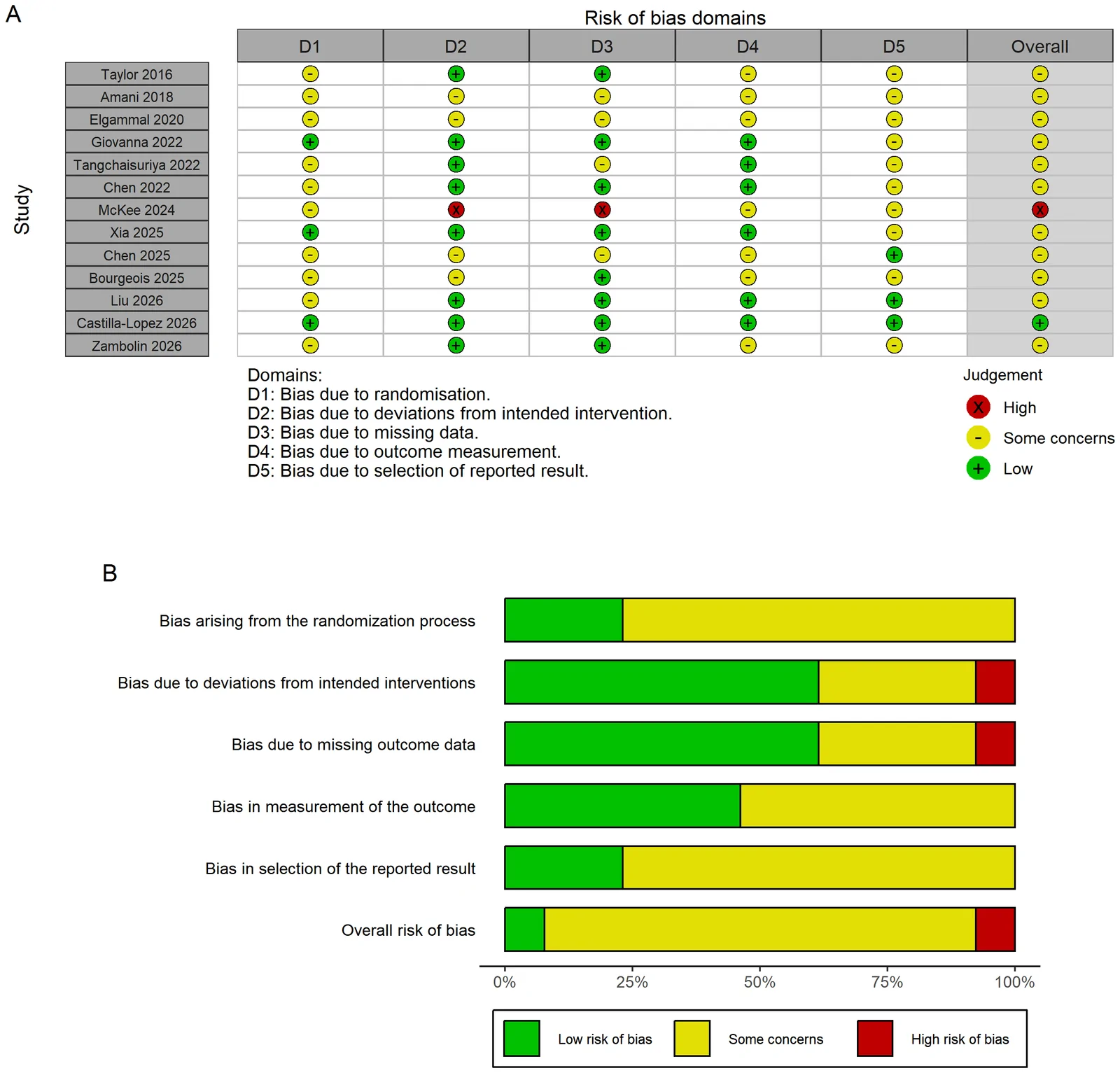
RoB 2 risk-of-bias summary. **(A)** Study-level risk-of-bias judgments across the five RoB 2 domains and overall risk for each included study. **(B)** Distribution of risk-of-bias judgments across each RoB 2 domain and overall risk. D1, bias arising from the randomization process; D2, bias due to deviations from intended interventions; D3, bias due to missing outcome data; D4, bias in measurement of the outcome; D5, bias in selection of the reported result. Green indicates low risk of bias, yellow indicates some concerns, and red indicates high risk of bias.

### 3.4 Meta-analysis

#### 3.4.1 Aerobic capacity

Eleven effect estimates from 11 independent studies contributed to the aerobic-capacity analysis (N = 253). The random-effects model showed a small benefit of BFR combined with interval training compared with non-BFR interval training (Hedges’ g = 0.30, 95% CI 0.05 to 0.55, p = 0.019). No between-study heterogeneity was observed for this outcome domain (I² = 0.0%).

#### 3.4.2 Anaerobic performance

For anaerobic performance, 11 effect estimates from 10 independent studies were included (N = 250). The pooled estimate also favored BFR combined with interval training, with a small effect size (Hedges’ g = 0.32, 95% CI 0.05 to 0.59, p = 0.022). Between-study heterogeneity was low (I² = 6.1%).

#### 3.4.3 Sport-specific performance

Eight effect estimates from 8 independent studies were available for sport-specific performance (N = 180). The pooled result favored BFR combined with interval training and indicated a small-to-moderate effect (Hedges’ g = 0.44, 95% CI 0.14 to 0.74, p = 0.004). No observed heterogeneity was detected in this analysis (I² = 0.0%). The main meta-analysis results are summarized in **Table 3**. Forest plots for aerobic capacity, anaerobic performance, and sport-specific performance are presented in **Figures 3**–**5**.

**Table 3:** Main analysis results.

Outcome	k	Hedges’ g	95% CI	p value	I² (%)	τ²	Model
Aerobic capacity	11	0.298	0.049 to 0.548	0.019	0.0	0.000	random effects REML
Anaerobic performance	11	0.317	0.047 to 0.587	0.022	6.1	0.021	random effects REML
Sport-specific performance	8	0.440	0.142 to 0.738	0.004	0.0	0.000	random effects REML

**Figure 3:**
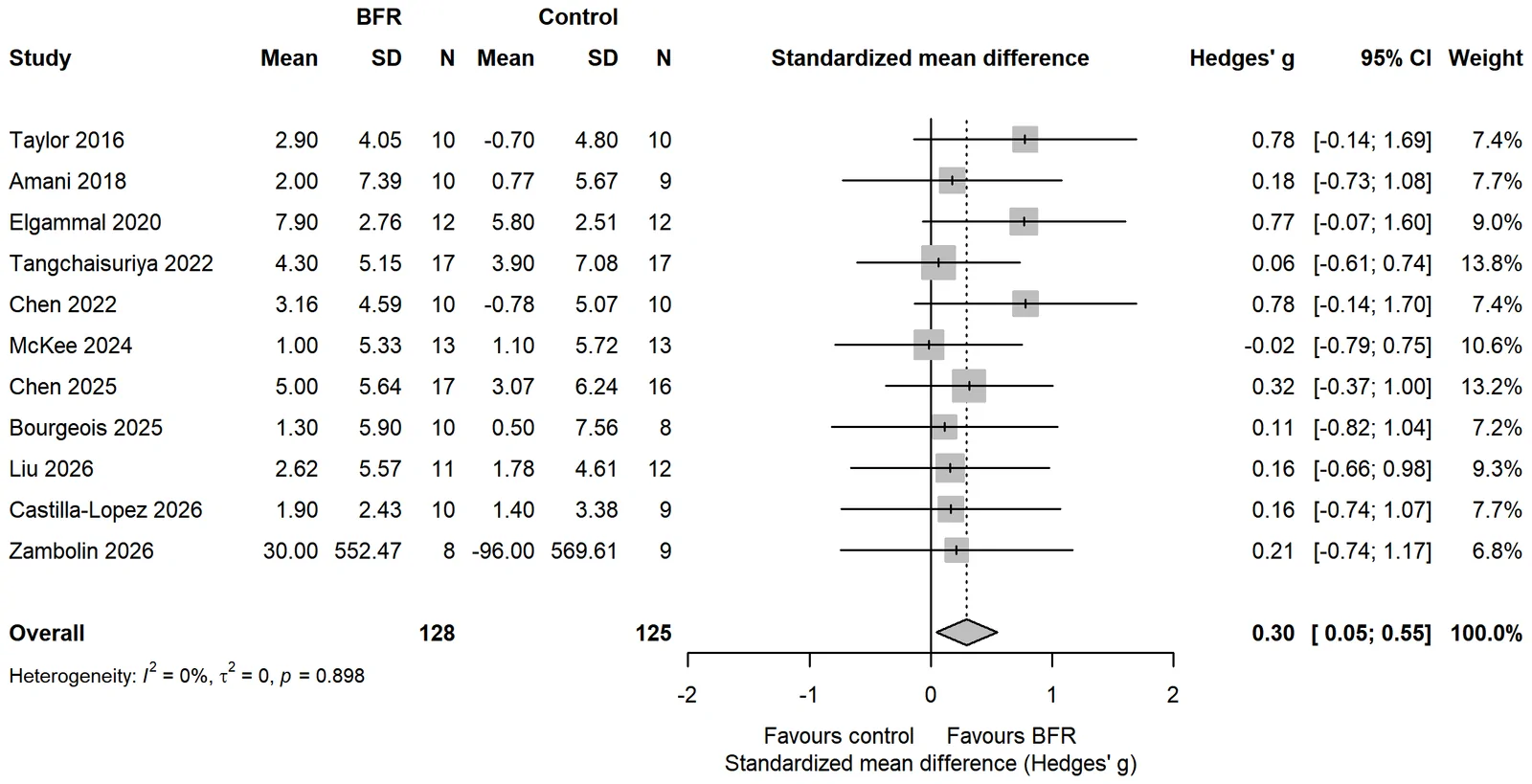
Forest plot for aerobic capacity. Values are Hedges’ g with 95% confidence intervals. Positive values favor BFR combined with interval training. The pooled effect was estimated using a random-effects REML model.

**Figure 4:**
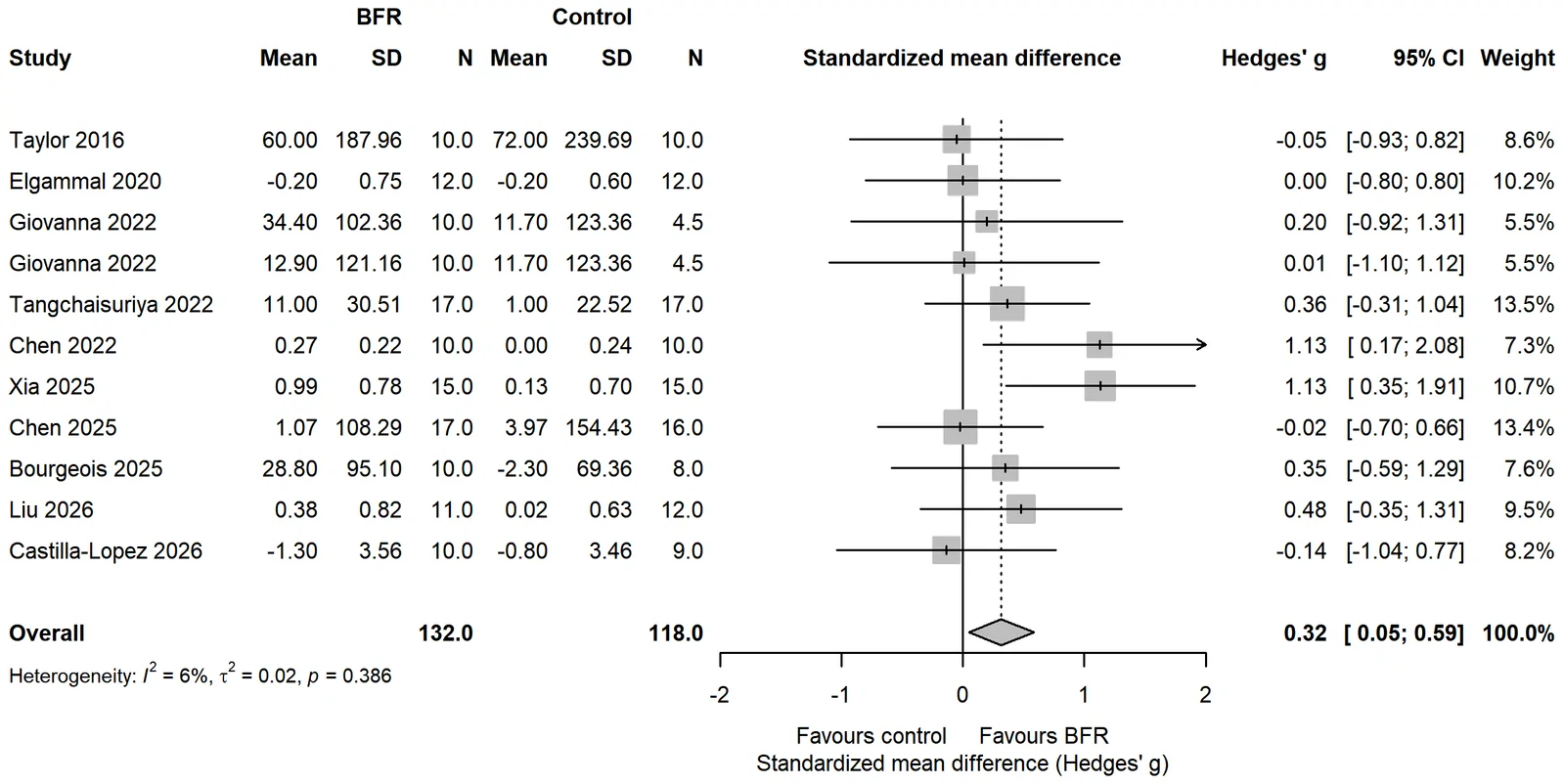
Forest plot for anaerobic performance. Values are Hedges’ g with 95% confidence intervals. Positive values favor BFR combined with interval training. The pooled effect was estimated using a random-effects REML model.

**Figure 5:**
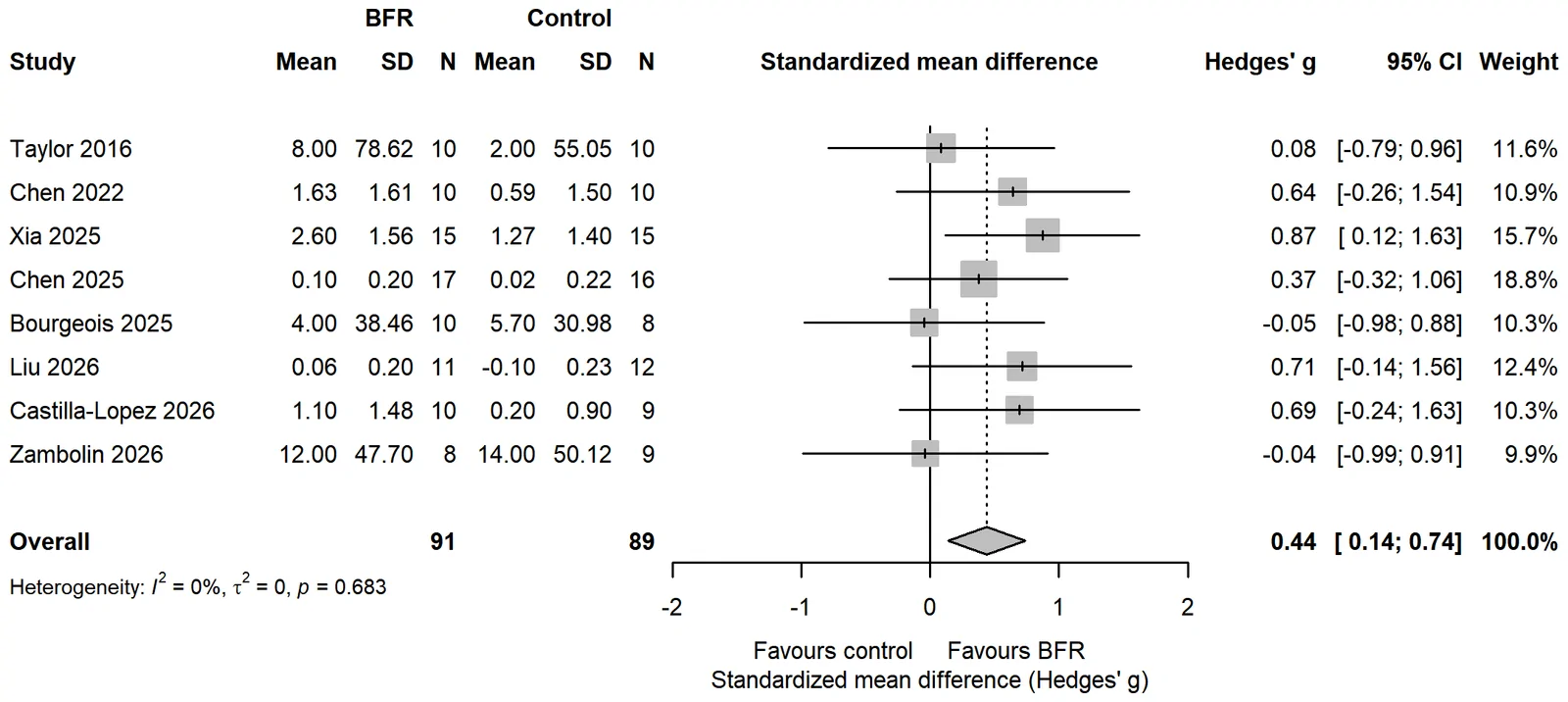
Forest plot for sport-specific performance. Values are Hedges’ g with 95% confidence intervals. Positive values favor BFR combined with interval training. The pooled effect was estimated using a random-effects REML model.

### 3.5 Assessment of small-study effects and sensitivity analysis

Assessment of small-study effects was limited to outcome domains with at least 10 effect estimates. Egger’s regression test did not indicate evidence of funnel-plot asymmetry for aerobic capacity (p = 0.473) or anaerobic performance (p = 0.954). The corresponding funnel plots are presented in **Figures 6**, **7**. The sport-specific performance analysis included fewer than 10 effect estimates, so funnel-plot interpretation and Egger’s regression testing were not performed for this outcome domain.

**Figure 6:**
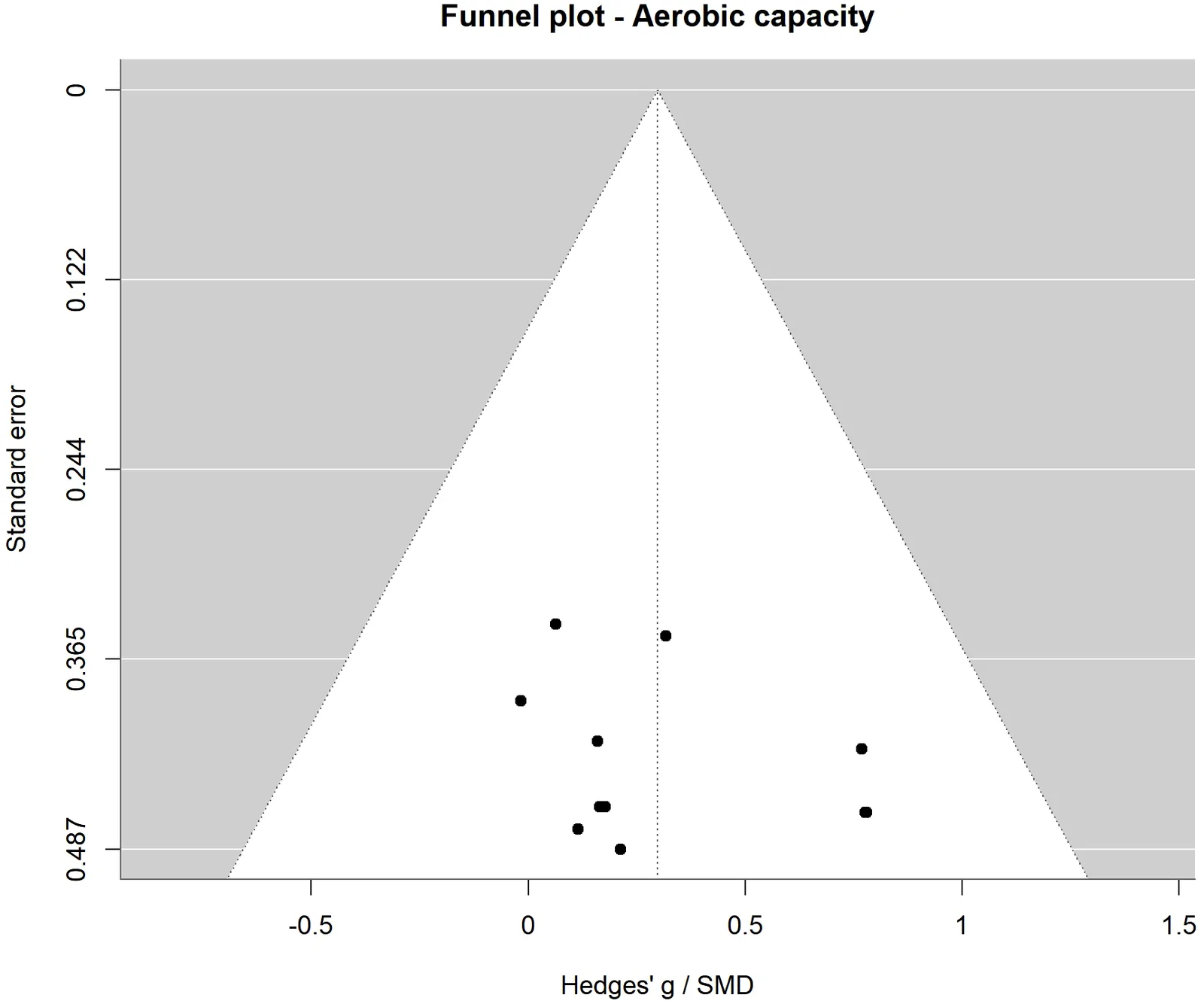
Funnel plot for aerobic capacity. The x-axis represents Hedges’ g/SMD and the y-axis represents standard error. Each point represents an individual effect estimate.

**Figure 7:**
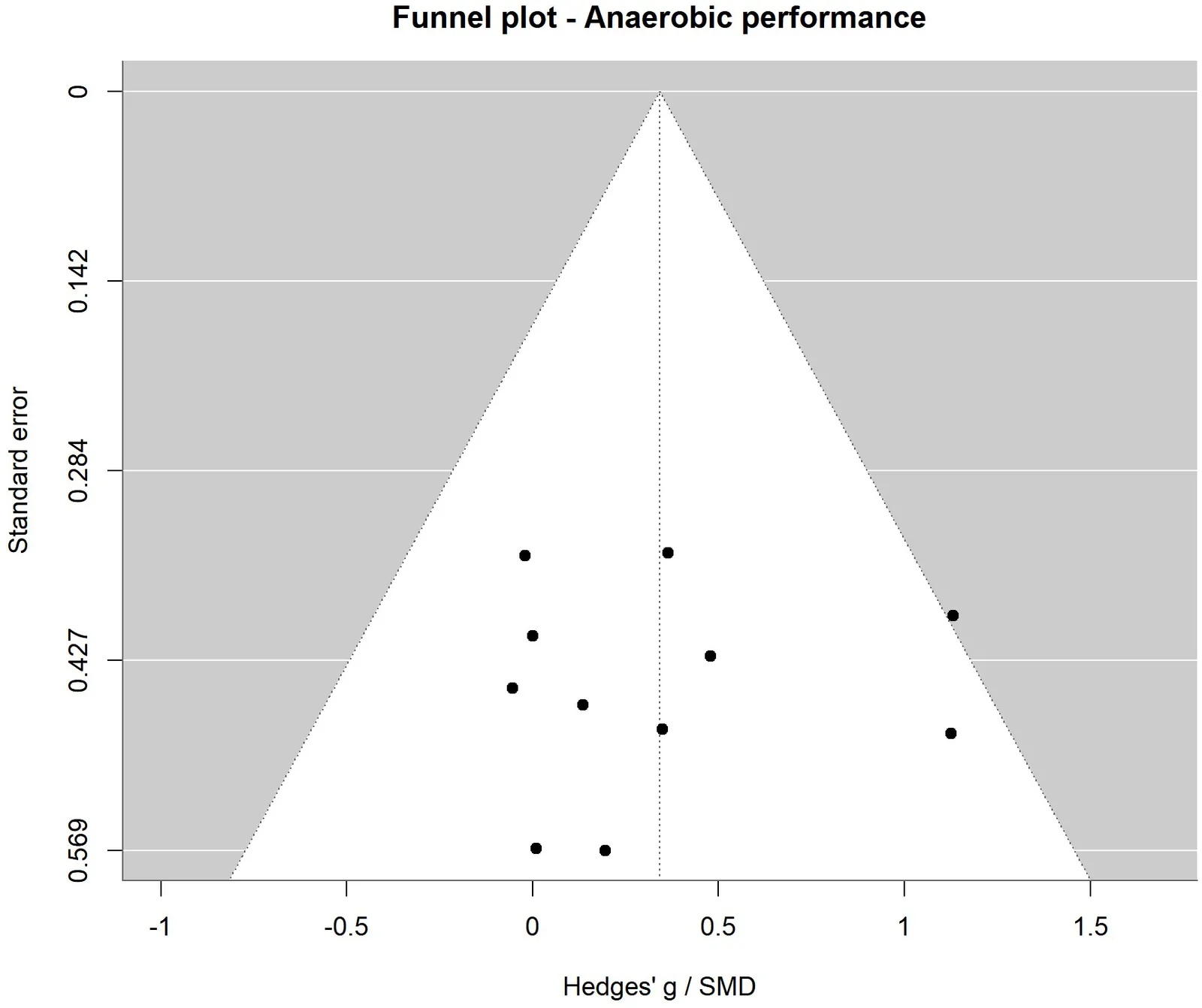
Funnel plot for anaerobic performance. The x-axis represents Hedges’ g/SMD and the y-axis represents standard error. Each point represents an individual effect estimate.

The prespecified sensitivity analysis produced estimates that remained consistent with the primary findings. Exclusion of McKee et al. (2024), the study judged to be at high risk of bias, affected only the aerobic-capacity analysis because this study did not contribute a selected primary effect estimate to the anaerobic-performance or sport-specific-performance analysis. After its exclusion, the pooled effect for aerobic capacity remained positive (Hedges’ g = 0.335, 95% CI 0.072 to 0.599, p = 0.013). In the shared-control sensitivity analysis for Giovanna et al. (2022), the anaerobic-performance estimate was similar whether only BFRG was retained (Hedges’ g = 0.362, 95% CI 0.085 to 0.639, p = 0.010) or only BFRrG was retained (Hedges’ g = 0.351, 95% CI 0.072 to 0.630, p = 0.014).

Alternative assumptions for the pre-post correlation used to estimate change-score SDs also led to comparable conclusions. When r = 0.3 was used, the pooled estimates remained positive for aerobic capacity (Hedges’ g = 0.257, 95% CI 0.008 to 0.505), anaerobic performance (Hedges’ g = 0.295, 95% CI 0.042 to 0.549), and sport-specific performance (Hedges’ g = 0.382, 95% CI 0.085 to 0.679). When r = 0.7 was used, the corresponding estimates were Hedges’ g = 0.371 for aerobic capacity (95% CI 0.120 to 0.622), Hedges’ g = 0.427 for anaerobic performance (95% CI 0.103 to 0.752), and Hedges’ g = 0.538 for sport-specific performance (95% CI 0.236 to 0.839). Across these sensitivity analyses, the direction of the primary effects was unchanged, although the magnitude and precision of the estimates varied across assumptions.

### 3.6 Subgroup analysis

Subgroup analysis did not identify evidence that the pooled effects differed by pressure prescription method, training frequency, or total planned training exposure. For pressure prescription method, subgroup-difference tests yielded p values of 0.134 for aerobic capacity, 0.487 for anaerobic performance, and 0.974 for sport-specific performance. The corresponding p values for training frequency were 0.788, 0.194, and 0.931, while those for total training sessions were 0.545, 0.481, and 0.169. These findings do not support interpreting subgroup-level point estimates as evidence that one pressure prescription method, training-frequency category, or total-session category was superior to another. The subgroup-difference test results are summarized in **Table 4**, and the full formal subgroup analysis results are provided in **Supplementary Table 3**.

**Table 4:** Moderator subgroup-difference tests.

Moderator	p for aerobic capacity	p for anaerobic performance	p for sport-specific performance
Pressure setting method	0.134	0.487	0.974
Training frequency	0.788	0.194	0.931
Total training sessions	0.545	0.481	0.169

BFR timing was assessed only as an exploratory subgroup variable because the distribution of BFR-t, BFR-c, and BFR-r comparisons was uneven. Subgroup-difference tests for BFR timing did not identify statistically significant differences for aerobic capacity (p = 0.552), anaerobic performance (p = 0.152), or sport-specific performance (p = 0.656). Given the unbalanced category distribution and limited number of comparisons, these results should be interpreted as hypothesis-generating rather than prescriptive.

### 3.7 Certainty of evidence

**Table 5** presents the certainty of evidence for the three primary outcomes assessed with the GRADE framework. The certainty of evidence was rated as low for aerobic capacity, anaerobic performance, and sport-specific performance. Across all three outcomes, downgrading was mainly driven by risk of bias and imprecision. Risk-of-bias concerns were present because most included studies were judged as having some concerns under RoB 2, and one study was judged to be at high risk of bias. Imprecision also contributed to downgrading, as the number of included studies and participants was limited and the confidence intervals did not allow precise estimation of the likely magnitude of effect.

**Table 5:** GRADE certainty of evidence for primary outcomes.

Outcome	Studies/effects	Participants	Risk of bias	Inconsistency	Indirectness	Imprecision	Publication bias	Certainty
Aerobic capacity	11 effects/11 studies	N = 253	Serious	Not serious, I² = 0.0%	Not serious	Serious	Not serious, Egger p = 0.473	Low
Anaerobic performance	11 effects/10 studies	N = 250	Serious	Not serious, I² = 6.1%	Not serious	Serious	Not serious, Egger p = 0.954	Low
Sport-specific performance	8 effects/8 studies	N = 180	Serious	Not serious, I² = 0.0%	Not serious	Serious	Not assessed, <10 effects	Low

GRADE certainty started at high because the evidence was derived primarily from randomized trials. Certainty was downgraded by one level for serious risk of bias when most studies contributing to an outcome were judged as having some concerns or high risk in RoB 2. Certainty was downgraded by one level for serious imprecision because the number of studies and participants was limited and confidence intervals did not allow precise estimation of clinically important effects. Indirectness was judged not serious for the evidence directly represented by the included studies, namely male trained athletes; generalizability to female athletes remains uncertain. Small-study effects were assessed using Egger’s test only when at least 10 effect estimates were available, and these tests have limited interpretability with small numbers of effects. GRADE, Grading of Recommendations Assessment, Development and Evaluation; CI, confidence interval; RoB 2, revised Cochrane risk-of-bias tool for randomized trials.

No further downgrading was applied for inconsistency because statistical heterogeneity was low in the primary analyses. Egger’s regression tests did not indicate evidence of funnel-plot asymmetry for aerobic capacity or anaerobic performance, although these tests should be interpreted cautiously given the limited number of effect estimates. Sport-specific performance included fewer than 10 effect estimates, so small-study effects were not formally assessed for this outcome.

## 4 Discussion

### 4.1 Principal findings

This systematic review and meta-analysis synthesized 15 reports from 13 independent studies examining BFR combined with interval training in trained athletes. Because all extracted comparisons involved male samples, the findings are directly applicable only to male trained athletes and should not be generalized to female athletes. Compared with identical or closely matched interval-training programs performed without BFR, adding BFR was associated with small, statistically significant additional improvements in aerobic capacity, anaerobic performance, and sport-specific performance. The primary models showed low statistical heterogeneity, while subgroup analysis did not identify evidence that pressure prescription method, training frequency, or total number of sessions clearly modified the pooled effects. Sensitivity analysis also left the direction of the estimates unchanged. This pattern suggests that BFR may provide a modest additional stimulus when integrated into interval training, although the strength of this inference remains limited because all three primary outcomes were rated as low-certainty evidence. The main contribution of this review is its specific comparison: it estimates the incremental effect of adding BFR to interval training, rather than the broader effect of BFR exercise compared with usual training, low-intensity training, or no training.

The practical meaning of these findings requires a more cautious reading. Statistical significance does not necessarily indicate an improvement large enough to matter in competition, particularly when pooled effects are small and the included studies do not consistently define smallest worthwhile changes, minimal important differences, or sport-specific performance thresholds. The available evidence therefore supports the possibility of a modest additive effect, but it does not yet establish that BFR-assisted interval training produces gains large enough to confer a meaningful competitive advantage. A formal comparison against a common smallest worthwhile change was not feasible because the included studies used heterogeneous outcomes and rarely reported sport- and test-specific SWC thresholds; consequently, the pooled standardized effects cannot be translated into a universal threshold of practical benefit.

### 4.2 Relationship to previous reviews

The present findings are broadly consistent with previous reviews suggesting that BFR exercise can improve selected physiological or performance-related outcomes, but the interpretation differs because the review question was narrower. Earlier reviews often pooled resistance, endurance, and mixed exercise modalities, or included both athletic and non-athletic populations (Slysz et al., 2016; Wortman et al., 2021). Reviews focused on endurance training, athlete performance, or BFR-HIIT have also reported potential benefits for aerobic or performance outcomes, while repeatedly noting substantial variation in participant characteristics, intervention protocols, and comparator conditions (Bennett and Slattery, 2019; Castilla-Lopez et al., 2022; Chua et al., 2022; Dong et al., 2025). Against that background, the modest effects observed in the present review are not unexpected. They may partly reflect the use of stricter active comparators, the narrower focus on interval-type training, and the already substantial training stimulus provided by interval training alone. Because the comparator condition was matched or closely matched interval training without BFR, the pooled estimates should be read as the added effect of BFR beyond interval training, rather than as the overall effect of introducing an exercise intervention.

### 4.3 Aerobic capacity

Aerobic capacity showed a small effect in favor of BFR combined with interval training (Hedges’ g = 0.30, 95% CI 0.05 to 0.55). This finding needs to be interpreted against the active-comparator design of the review. The comparison condition was not no training or usual training, but interval training performed without BFR; therefore, the pooled estimate reflects the added value of BFR on top of an already potent training stimulus. This distinction is important because HIIT and SIT alone are well-established methods for improving VO_2_max and endurance-related adaptation (Laursen and Jenkins, 2002; Milanovic et al., 2015; MacInnis and Gibala, 2017). A small pooled effect under this comparator structure may therefore still be meaningful from a training-design perspective, but it should not be interpreted as the total aerobic benefit of interval training itself.

The physiological explanation is plausible, although it remains indirect in the present review. Adding BFR to interval training may increase local hypoxia and metabolic stress during exercise, thereby strengthening the peripheral stimulus imposed on the working muscles (Patterson et al., 2019; Chua et al., 2022). In endurance-trained athletes, this added stress could interact with oxygen-utilization, vascular, and mitochondrial adaptations that are relevant to aerobic performance (Ferguson et al., 2021). Even so, VO_2_max is constrained by both central and peripheral determinants, and the present findings should not be reduced to a single BFR-related mechanism (Bassett and Howley, 2000). This caution is especially relevant because the aerobic-capacity analysis drew on heterogeneous interval formats, including sprint interval training, interval running, HIIT, repeated-sprint or sport-specific interval models, and cycling-based protocols (Taylor et al., 2016; Amani et al., 2018; Elgammal et al., 2020; Tangchaisuriya et al., 2022; Chen et al., 2022a, b). Taken together, the evidence supports a small additive effect on aerobic capacity, but it does not yet establish that BFR-assisted interval training consistently produces practically or competitively meaningful improvements in VO_2_max or endurance performance across male trained athletes.

### 4.4 Anaerobic performance

Anaerobic performance showed a small effect in favor of BFR combined with interval training (Hedges’ g = 0.32, 95% CI 0.05 to 0.59). The direction of the estimate is consistent with a possible additive training stimulus, but the magnitude was limited and the certainty of evidence was low. This result should therefore be read as preliminary evidence across a broad high-intensity performance domain, rather than as direct proof of a specific anaerobic adaptation.

This caution is important because the outcome domain was not physiologically uniform. The analysis included Wingate peak and mean power, cycling peak power output or force-velocity Pmax, repeated-sprint outcomes, shuttle- or suicide-run performance, and strength-dominant measures such as isokinetic knee extensor strength. These tests are related in that they all involve short-duration, high-intensity effort, but they do not assess the same underlying construct. Wingate-derived outcomes and force-velocity tests capture different aspects of short-term power production, whereas field-based shuttle tests and strength measures introduce movement-specific, coordination, fatigue-resistance, and neuromuscular components (Bar-Or, 1987; Vandewalle et al., 1987). Energy-system contribution also changes continuously with exercise duration and intensity, making it inappropriate to treat all short-duration outcomes as interchangeable indicators of a single physiological pathway (Gastin, 2001).

Repeated-sprint and shuttle-based outcomes add another layer of complexity. Performance in these tests depends not only on initial sprint capacity, but also on phosphocreatine resynthesis, glycolytic contribution, oxidative recovery between efforts, fatigue tolerance, and the ability to reproduce neuromuscular output under repeated stress (Spencer et al., 2005; Girard et al., 2011). This is why repeated-sprint training recommendations place considerable emphasis on work-rest structure, recovery mode, and sport-specific movement demands (Bishop et al., 2011). These tests are highly relevant to intermittent sports, but they should not be interpreted in the same way as laboratory-based peak-power tests.

A mechanistic explanation is plausible, although it remains indirect. BFR may increase local hypoxia, metabolite accumulation, and internal load during interval exercise, thereby adding metabolic stress to an already demanding training stimulus (Suga et al., 2012; Patterson et al., 2019; Chua et al., 2022). Greater metabolic stress, however, does not automatically produce greater external mechanical output. In some acute settings, BFR can increase physiological strain while reducing muscle activation or mechanical performance (Teixeira et al., 2018). The small pooled effect observed in this review may therefore reflect a balance between added internal stress and the already strong anaerobic and neuromuscular stimulus provided by interval training alone.

The response to BFR combined with interval training is likely to depend on how the session is programed, including sprint duration, interval structure, work-rest ratio, recovery mode, exercise modality, and total training exposure. These variables are central to high-intensity interval training prescription, especially when the target is anaerobic or neuromuscular performance (Buchheit and Laursen, 2013b). In the present review, subgroup analysis did not identify clear evidence that training frequency, total number of sessions, pressure prescription, or BFR timing modified the pooled effect. Given the limited number of studies and the heterogeneity of outcomes, this should not be interpreted as evidence that these variables are unimportant. Future meta-analyses should separate Wingate-derived power, repeated-sprint ability, field-based high-intensity performance tests, and strength-dominant outcomes once enough studies become available.

### 4.5 Sport-specific performance

Sport-specific performance showed the numerically largest point estimate among the three primary outcome domains (Hedges’ g = 0.44, 95% CI 0.14 to 0.74). This finding should not be read as stronger evidence simply because the point estimate was larger. The analysis was based on only eight effect estimates, the certainty of evidence was rated as low, and the included tests captured different forms of sport-related performance. The pooled result therefore provides preliminary evidence that BFR combined with interval training may transfer to selected performance tests, but it does not establish that this approach improves actual competitive performance.

The interpretation of this domain depends heavily on what is meant by “sport-specific performance.” A test can be considered practically useful only when it reflects the demands of the target sport, shows acceptable reliability, and is sensitive enough to detect changes that matter in practice rather than random variation (Currell and Jeukendrup, 2008; Stevens and Dascombe, 2015). This distinction is especially important in trained athletes, where small statistical effects may not exceed the smallest worthwhile change for a given event, test, or competitive context (Hopkins et al., 2009). For this reason, the present domain should be understood as a collection of sport-related performance measures rather than as a single uniform construct.

Transfer from BFR-assisted interval training is likely to depend on how closely the training stimulus matches the outcome test. Repeated-sprint and high-intensity intermittent tests may align more directly with repeated-sprint training, small-sided games, and other intermittent protocols. Time-trial, running, and cycling outcomes, by contrast, may depend more on sustained power production, speed regulation, and pacing behavior. Field-based sport-specific tests can improve ecological relevance, but they may also incorporate technical execution, tactical decision-making, motivation, and familiarity with the testing environment, making it difficult to isolate physiological adaptation alone. Longer time-trial outcomes introduce further complexity because performance may be shaped by pacing strategy and by the interaction between central and peripheral fatigue, both of which vary with task duration and intensity (Abbiss and Laursen, 2008; Thomas et al., 2015).

The studies contributing to this domain included cycling time trials, running performance tests, repeated-sprint ability, badminton-specific performance, basketball small-sided-game contexts, soccer interval-running outcomes, and cycling HIIT protocols (Taylor et al., 2016; Chen et al., 2022a; Chen et al., 2025; Xia et al., 2025; Bourgeois et al., 2025; Liu et al., 2026; Castilla-Lopez et al., 2026; Zambolin et al., 2026). Previous reviews of BFR in high-performance athletes and BFR-HIIT support the possibility that BFR may be useful as an adjunct in selected high-intensity or high-performance settings, but they also emphasize substantial protocol heterogeneity and unresolved evidence gaps (Pignanelli et al., 2021; Chua et al., 2022). Taken together, the current evidence suggests that BFR may improve selected sport-related performance tests when the training and testing demands are closely aligned. It does not yet support a broader claim that BFR-assisted interval training enhances competition performance across sports.

### 4.6 Moderator variables and BFR prescription implications

Subgroup analysis did not identify clear evidence that the pooled effects were moderated by pressure prescription method, training frequency, or total number of training sessions. Some subgroup point estimates were numerically larger than others, but the subgroup-difference tests did not support a conclusion that any pressure prescription method, weekly frequency category, or total-session category was superior. BFR application timing was treated only as an exploratory variable because BFR-t, BFR-c, and BFR-r comparisons were unevenly distributed, with some categories represented by only one or two effects. These subgroup findings should therefore be interpreted as hypothesis-generating rather than prescriptive.

The lack of statistically significant subgroup differences should not be mistaken for evidence that BFR prescription variables are unimportant. These analyses were limited by small numbers of studies, uneven category distributions, and the use of study-level classifications; they do not provide individual-level dose-response evidence. This distinction matters because BFR pressure is not determined by the nominal pressure value alone. The degree of vascular restriction can vary according to cuff width, cuff material, limb circumference, and whether pressure is prescribed relative to arterial or limb occlusion pressure (Loenneke et al., 2012, 2013). A fixed absolute pressure may therefore produce different physiological constraints across athletes, even when the same mmHg value is used. Recent BFR reporting recommendations emphasize transparent and detailed reporting of the apparatus and methodology used, including device characteristics, cuff properties, pressure measurement and regulation, and the timing of cuff inflation and deflation (Hughes et al., 2025). Earlier methodological guidance also supports individualized pressure prescription where feasible (Scott et al., 2015; Patterson et al., 2019). Based on the current evidence, no firm recommendation can be made regarding an optimal BFR pressure, timing strategy, weekly frequency, or total session dose for male trained athletes. Given the substantial variation in pressure-setting methods, cuff characteristics, application timing, and interval-training protocols, standardization of an optimal BFR interval-training protocol is currently not possible. Future trials should, at minimum, report the BFR device and cuff characteristics, cuff width and material, the method used to determine AOP or LOP, the body position used during occlusion-pressure assessment, the target and applied pressure, pressure regulation, and the timing and duration of cuff inflation and deflation, together with session tolerability.

### 4.7 Safety and practical application

The included interventions used several approaches to BFR pressure prescription, including fixed mmHg pressure, progressive fixed pressure, systolic-blood-pressure-based pressure, %AOP, and %LOP. This variation is important for interpretation because adverse events, discomfort, and tolerability were not consistently reported or synthesized as formal outcomes. As a result, the present review cannot draw a strong safety conclusion. The inconsistent reporting of adverse events and session tolerability constitutes a major limitation of the current evidence base and leaves the safety profile of BFR combined with high-intensity interval training uncertain. The absence of consistently reported adverse events should not be interpreted as evidence that BFR is risk-free, particularly when it is combined with high-intensity interval formats. More recent BFR risk-stratification frameworks emphasize structured pre-participation screening based on relevant medical history, cardiovascular and vascular conditions, thrombotic risk factors, and signs or symptoms that may warrant further medical evaluation before BFR exposure (Nascimento et al., 2022). Athlete-focused guidance similarly supports structured pre-screening and careful pressure determination before BFR is incorporated into training (Gaviglio et al., 2026). Recent experimental studies have also broadened safety assessment beyond the simple occurrence of adverse events by incorporating cardiovascular and perceptual responses, including blood pressure, heart rate, perceived discomfort and exertion, as well as vascular measures such as arterial stiffness (Jacobs et al., 2023; Rolnick et al., 2024). These developments support prospective and standardized safety monitoring rather than inferring safety from the absence of reported adverse events.

For the male trained athletes represented in the available studies, BFR is best viewed as an adjunctive loading strategy rather than a substitute for well-designed sport training. This is consistent with an integrated, periodized approach to athlete preparation (Mujika et al., 2018). Its practical appeal lies in the possibility of increasing internal hypoxic and metabolic stress without a proportional increase in external mechanical load, which may be useful during selected training phases or return-to-performance progressions (Wortman et al., 2021; Ferguson et al., 2021). Practical use should remain conditional on structured pre-participation risk screening, individualized pressure prescription where possible, and close monitoring of session tolerance. Monitoring should include adverse symptoms and events, pain or discomfort, perceived exertion, and, where appropriate, cardiovascular or vascular responses, alongside cuff placement, pressure setting, total session load, and the athlete’s existing training burden.

### 4.8 Limitations

Several limitations should be considered. First, confidence in the pooled estimates was limited by the small evidence base and risk-of-bias concerns. Only 15 reports representing 13 independent studies were included, with limited participant numbers within each outcome domain. Most studies were judged as having some concerns under RoB 2, one study was judged as high risk, and GRADE certainty was low for all three primary outcomes. Therefore, the magnitude and practical relevance of the observed effects remain uncertain.

Second, generalizability and prescription-level interpretation are limited. All extracted comparisons involved male athletes, so the findings should not be extended to female athletes without direct evidence. The included studies varied in sport, interval format, cuff width, cuff position, pressure prescription, and BFR timing, preventing firm conclusions about the optimal BFR prescription. Outcome heterogeneity also limits interpretation, particularly for anaerobic and sport-specific performance, where pooled measures captured different constructs and levels of ecological validity. Third, adverse events, discomfort, and session tolerability were inconsistently reported. This represents a major limitation of the current review and prevents reliable characterization of the safety profile of BFR combined with interval training. Finally, some change-score SDs were estimated using assumed pre-post correlations, small-study effects could not be formally assessed for sport-specific performance, and the English-language restriction may have introduced selection bias. Low I² values should therefore not be interpreted as evidence of clinical or methodological homogeneity.

## 5 Conclusion and future research recommendations

In trained male athletes, BFR combined with interval training was associated with small positive effects on aerobic capacity, anaerobic performance, and sport-specific performance. However, the low certainty of evidence, uncertain practical significance, heterogeneous prescription methods, and incomplete safety reporting preclude recommending this approach as a primary performance-enhancement strategy. It should be considered only as a cautiously monitored adjunct in selected training contexts.

Future research should use adequately powered, preregistered, sport-specific randomized trials with matched active comparators. This design is needed to separate the incremental effect of BFR from the effect of interval training itself. Trials should report BFR prescription in sufficient detail, preferably using individualized pressure methods where feasible, and should include cuff width, cuff material, cuff position, limb size, pressure progression, and the method used to determine occlusion pressure. Future trials should prospectively report BFR-specific pre-participation screening criteria and systematically monitor adverse events, withdrawals attributable to BFR, pain or discomfort, tolerability, perceived exertion, and, where appropriate, cardiovascular and vascular safety measures. The evidence base also needs studies involving female athletes, broader competitive levels, and a wider range of sports. Future trials should use reliable and harmonized aerobic, anaerobic, and sport-specific performance outcomes so that later meta-analyses can better distinguish true training effects from measurement error and construct heterogeneity.
