# Supplementary material for: Effects of blood flow restriction combined with interval training on aerobic capacity, anaerobic performance, and sport-specific performance in trained male athletes: a systematic review and meta-analysis
Source: Front Physiol. 2026 Sep 16;17:1928418. doi: 10.3389/fphys.2026.1928418 (PMC13624964; doi:10.3389/fphys.2026.1928418)
Supplement: Supplementary file 1 [file SupplementaryFile1.docx]

**Supplementary File 1. Complete database search strategies**

**Search date: 15 May 2026**

1. PubMed

(("Blood Flow Restriction Therapy"[Mesh] OR "blood flow restriction"[tiab] OR "blood-flow restriction"[tiab] OR "blood flow restricted"[tiab] OR "blood-flow restricted"[tiab] OR "blood flow restriction training"[tiab] OR "blood flow restriction exercise"[tiab] OR BFR[tiab] OR "BFR training"[tiab] OR KAATSU[tiab] OR "occlusion training"[tiab] OR "occlusion exercise"[tiab] OR "vascular occlusion"[tiab] OR "partial vascular occlusion"[tiab])

AND

("High-Intensity Interval Training"[Mesh] OR "interval training"[tiab] OR "interval exercise"[tiab] OR "intermittent training"[tiab] OR "intermittent exercise"[tiab] OR "high intensity interval training"[tiab] OR "high-intensity interval training"[tiab] OR HIIT[tiab] OR "sprint interval training"[tiab] OR SIT[tiab] OR "repeated sprint"[tiab] OR "repeated-sprint"[tiab] OR "repeated sprint training"[tiab] OR "repeated-sprint training"[tiab] OR "interval running"[tiab] OR "interval cycling"[tiab])

AND

(athlete*[tiab] OR trained[tiab] OR competitive[tiab] OR elite[tiab] OR professional[tiab]))

2. Web of Science

TS=("blood flow restriction" OR "blood-flow restriction" OR "blood flow restricted" OR "blood-flow restricted" OR "blood flow restriction training" OR "blood flow restriction exercise" OR BFR OR "BFR training" OR KAATSU OR "occlusion training" OR "occlusion exercise" OR "vascular occlusion" OR "partial vascular occlusion")

AND

TS=("interval training" OR "interval exercise" OR "intermittent training" OR "intermittent exercise" OR "high intensity interval training" OR "high-intensity interval training" OR HIIT OR "sprint interval training" OR SIT OR "repeated sprint" OR "repeated-sprint" OR "repeated sprint training" OR "repeated-sprint training" OR "interval running" OR "interval cycling")

AND

TS=(athlete* OR trained OR competitive OR elite OR professional)

3. Scopus

TITLE-ABS-KEY(("blood flow restriction" OR "blood-flow restriction" OR "blood flow restricted" OR "blood-flow restricted" OR "blood flow restriction training" OR "blood flow restriction exercise" OR BFR OR "BFR training" OR KAATSU OR "occlusion training" OR "occlusion exercise" OR "vascular occlusion" OR "partial vascular occlusion")

AND

("interval training" OR "interval exercise" OR "intermittent training" OR "intermittent exercise" OR "high intensity interval training" OR "high-intensity interval training" OR HIIT OR "sprint interval training" OR SIT OR "repeated sprint" OR "repeated-sprint" OR "repeated sprint training" OR "repeated-sprint training" OR "interval running" OR "interval cycling")

AND

(athlete* OR trained OR competitive OR elite OR professional))

4. SPORTDiscus via EBSCO

TX (("blood flow restriction" OR "blood-flow restriction" OR "blood flow restricted" OR "blood-flow restricted" OR "blood flow restriction training" OR "blood flow restriction exercise" OR BFR OR "BFR training" OR KAATSU OR "occlusion training" OR "occlusion exercise" OR "vascular occlusion" OR "partial vascular occlusion")

AND

("interval training" OR "interval exercise" OR "intermittent training" OR "intermittent exercise" OR "high intensity interval training" OR "high-intensity interval training" OR HIIT OR "sprint interval training" OR SIT OR "repeated sprint" OR "repeated-sprint" OR "repeated sprint training" OR "repeated-sprint training" OR "interval running" OR "interval cycling")

AND

(athlete* OR trained OR competitive OR elite OR professional))

5. Embase

(('blood flow restriction' OR 'blood-flow restriction' OR 'blood flow restricted' OR 'blood-flow restricted' OR 'blood flow restriction training' OR 'blood flow restriction exercise' OR bfr OR 'BFR training' OR kaatsu OR 'occlusion training' OR 'occlusion exercise' OR 'vascular occlusion' OR 'partial vascular occlusion'):ti,ab,kw)

AND

(('interval training' OR 'interval exercise' OR 'intermittent training' OR 'intermittent exercise' OR 'high intensity interval training' OR 'high-intensity interval training' OR hiit OR 'sprint interval training' OR sit OR 'repeated sprint' OR 'repeated-sprint' OR 'repeated sprint training' OR 'repeated-sprint training' OR 'interval running' OR 'interval cycling'):ti,ab,kw)

AND

((athlete* OR trained OR competitive OR elite OR professional):ti,ab,kw)

6. Cochrane Library

#1 MeSH descriptor: [Blood Flow Restriction Therapy] explode all trees

#2 ("blood flow restriction" OR "blood-flow restriction" OR "blood flow restricted" OR "blood-flow restricted" OR "blood flow restriction training" OR "blood flow restriction exercise" OR BFR OR "BFR training" OR KAATSU OR "occlusion training" OR "occlusion exercise" OR "vascular occlusion" OR "partial vascular occlusion"):ti,ab,kw

#3 #1 OR #2

#4 MeSH descriptor: [High-Intensity Interval Training] explode all trees

#5 ("interval training" OR "interval exercise" OR "intermittent training" OR "intermittent exercise" OR "high intensity interval training" OR "high-intensity interval training" OR HIIT OR "sprint interval training" OR SIT OR "repeated sprint" OR "repeated-sprint" OR "repeated sprint training" OR "repeated-sprint training" OR "interval running" OR "interval cycling"):ti,ab,kw

#6 #4 OR #5

#7 (athlete* OR trained OR competitive OR elite OR professional):ti,ab,kw

#8 #3 AND #6 AND #7
