# Supplementary material for: Effects of blood flow restriction combined with interval training on aerobic capacity, anaerobic performance, and sport-specific performance in trained male athletes: a systematic review and meta-analysis
Source: Front Physiol. 2026 Sep 16;17:1928418. doi: 10.3389/fphys.2026.1928418 (PMC13624964; doi:10.3389/fphys.2026.1928418)
Supplement: Supplementary file 2 [file Table2.docx]

**Supplementary Table 2. Full intervention protocols and BFR parameters**

| **Study** | **Interval type** | **BFR group** | **Comparator** | **BFR timing** | **Duration** | **Frequency** | **Sessions** | **Exercise intensity** | **Cuff position** | **Cuff width** | **Pressure method** | **Pressure** | **Outcomes** |
| --- | --- | --- | --- | --- | --- | --- | --- | --- | --- | --- | --- | --- | --- |
| Taylor 2016 | sprint interval training | SIT + postexercise BFR | SIT alone | During recovery/post-exercise | 4 weeks | 2 sessions/week | 8 | Repeated 30-s maximal sprint cycling | proximal portion of each thigh | NR | Fixed absolute pressure | 130 mmHg | Aerobic capacity: VO₂max; Anaerobic performance: PPO during 30s maximal sprint cycling; Sport-specific performance: 15km time trial time |
| Amani 2018 | interval running | Interval training with Kaatsu/BFR | Normal interval training without Kaatsu | During exercise | 2 weeks | 4 sessions/week | 8 | 60-70% maximum heart rate reserve | upper section of thigh | NR | Fixed absolute pressure | 140-180 mmHg | Aerobic capacity: VO₂max |
| Elgammal 2020 | repeated-sprint training | Repeated sprint training with Kaatsu/BFR | Repeated sprint training without BFR | During exercise | 4 weeks | 3 sessions/week | 12 | Maximal repeated shuttle sprints | proximal region of both legs | NR | Progressive fixed absolute pressure | 100-160 mmHg | Aerobic capacity: 20m shuttle estimated VO₂max; Anaerobic performance: Suicide Run |
| Giovanna 2022 | repeated-sprint training | Repeated sprint training with BFR during exercise | Repeated sprint training in normal condition without BFR | During exercise | 2 weeks | 3 sessions/week | 6 | 10-s maximal cycling sprints | lower limbs / proximal thigh cuffs | cuff size 11 × 85 cm; bladder 10 × 41 cm | %AOP-based | 45% AOP; 88.2 ± 10.1 mmHg | Anaerobic performance: Force velocity Pmax |
| Giovanna 2022 | repeated-sprint training | Repeated sprint training with BFR during recovery | Repeated sprint training in normal condition without BFR | During recovery/post-exercise | 2 weeks | 3 sessions/week | 6 | 10-s maximal cycling sprints | lower limbs / proximal thigh cuffs | cuff size 11 × 85 cm; bladder 10 × 41 cm | %AOP-based | 45% AOP; 88.2 ± 10.1 mmHg | Anaerobic performance: Force velocity Pmax |
| Tangchaisuriya 2022 | HIIT | Continuous training + HIIT with BFR | Continuous training + HIIT without BFR | During exercise | 12 weeks | 2 lab HIIT/BFR sessions/week | 24 | HIIT bouts at 80% PPO; BFRIT second/fourth bouts at 60% PPO | proximal thighs | 11-cm-wide pneumatic cuff | %AOP-based | 30% AOP | Aerobic capacity: VO₂max; Anaerobic performance: Isokinetic knee extension peak torque |
| Chen 2022 | interval running | Running interval training with BFR | Running interval training without BFR | Continuous | 8 weeks | 3 sessions/week | 24 | 50% heart rate reserve | most proximal portion of each thigh | 14.2 × 67.5 cm | SBP-based | 1.3 × resting SBP; 154 ± 6 mmHg | Aerobic capacity: VO₂max; Anaerobic performance: Isokinetic knee extensor strength at 180 deg per s; Sport-specific performance: Maximal running performance |
| McKee 2024/2025 | repeated-sprint training | Cycling repeated-sprint training with BFR | Cycling repeated-sprint training without BFR | Continuous | 3 weeks | 3 sessions/week | 9 | 5-s maximal cycling sprints | most proximal portion of the legs | 10 cm | %AOP-based | 45% AOP; 72 (6) mmHg | Aerobic capacity: VO₂max |
| Xia 2025 | Tabata | BFR-Tabata | Tabata without effective pressure | During exercise | 6 weeks | 3 sessions/week | 18 | 20-s maximal exercise / 10-s recovery Tabata | upper thigh, about 1-2 cm distal to inguinal crease | 5 cm | %AOP-based | 60% AOP | Anaerobic performance: Wingate peak power; Sport-specific performance: Badminton specific endurance test |
| Chen 2025 | HIIT/MIIT | BFR moderate-intensity interval training | High-intensity interval training without BFR | During exercise | 6 weeks | 2 sessions/week | 12 | BFR-MIIT 70-75% HRmax; HIIT 85-95% HRmax | lower extremities | NR | Fixed absolute pressure | 180-200 mmHg | Aerobic capacity: 20m shuttle estimated VO₂max; Anaerobic performance: Wingate peak power; Sport-specific performance: 30m sprint |
| Bourgeois 2025 | HIIT | HIIT with BFR | HIIT without BFR | During exercise | 3 weeks | 3 sessions/week | 9 | 15-s efforts at 100% MAP, 15-s recovery at 60 W | most proximal part of both thighs | 9 cm | %AOP-based | 50-70% AOP | Aerobic capacity: VO₂peak; Anaerobic performance: Wingate mean power; Sport-specific performance: 5km time trial time |
| Liu 2026 | small-sided games | 3 vs 3 small-sided games with BFR | 3 vs 3 small-sided games without BFR | During exercise | 4 weeks | 2 sessions/week | 8 | 3 vs 3 basketball SSG, progressive duration | bilateral proximal thighs | 10 cm | SBP-based | 100-130% estimated leg SBP | Aerobic capacity: VO₂peak; Anaerobic performance: Wingate mean power; Sport-specific performance: RSA best time |
| Castilla-Lopez 2026 | interval running | Interval running with BFR | Interval running without BFR | During exercise | 6 weeks | 2 sessions/week | 12 | 50% MAS, increasing by 5% every four sessions | most proximal part of the leg, below the gluteal line | 10 cm; cuff length 44.5-58.5 cm for 95% | %AOP-based | 60% AOP | Aerobic capacity: YoYo IR1 estimated VO₂max; Anaerobic performance: RSA decrement score; Sport-specific performance: Vmax |
| Zambolin 2026 | HIIT | Effort-matched HIIT with BFR | Effort-matched HIIT without BFR | During exercise | 6-day microcycle | 5 sessions/6 days | 5 | RPE 17-19; 6 × 5-min intervals | proximal part of the thighs | 13 cm | %LOP-based | 180-220 mmHg; about 80% LOP | Aerobic capacity: VO₂max; Sport-specific performance: MPO 5min all out |

Note. NR, not reported; AOP, arterial occlusion pressure; LOP, limb occlusion pressure; SBP, systolic blood pressure.
