# Supplementary material for: Effects of blood flow restriction combined with interval training on aerobic capacity, anaerobic performance, and sport-specific performance in trained male athletes: a systematic review and meta-analysis
Source: Front Physiol. 2026 Sep 16;17:1928418. doi: 10.3389/fphys.2026.1928418 (PMC13624964; doi:10.3389/fphys.2026.1928418)
Supplement: Supplementary file 3 [file Table3.docx]

**Supplementary Table 3. Full formal subgroup analysis results**

| **Outcome** | **Subgroup** | **Level** | **k** | **Hedges' g [95% CI]** | **p value** | **I² (%)** | **Subgroup p** |
| --- | --- | --- | --- | --- | --- | --- | --- |
| Aerobic capacity | Pressure setting method | Non-occlusion-based pressure | 6 | 0.474 [0.135, 0.812] | 0.006 | 0.0 | 0.134 |
| Aerobic capacity | Pressure setting method | Occlusion-based pressure | 5 | 0.092 [-0.276, 0.460] | 0.626 | 0.0 | 0.134 |
| Anaerobic performance | Pressure setting method | Non-occlusion-based pressure | 5 | 0.250 [-0.137, 0.638] | 0.205 | 11.6 | 0.487 |
| Anaerobic performance | Pressure setting method | Occlusion-based pressure | 6 | 0.436 [0.075, 0.797] | 0.018 | 1.8 | 0.487 |
| Sport-specific performance | Pressure setting method | Non-occlusion-based pressure | 4 | 0.445 [0.039, 0.852] | 0.032 | 0.0 | 0.974 |
| Sport-specific performance | Pressure setting method | Occlusion-based pressure | 4 | 0.421 [-0.070, 0.911] | 0.093 | 19.3 | 0.974 |
| Aerobic capacity | Training frequency | <=2 sessions/week | 5 | 0.265 [-0.083, 0.613] | 0.135 | 0.0 | 0.788 |
| Aerobic capacity | Training frequency | >=3 sessions/week | 6 | 0.334 [-0.024, 0.691] | 0.067 | 0.0 | 0.788 |
| Anaerobic performance | Training frequency | <=2 sessions/week | 5 | 0.185 [-0.162, 0.532] | 0.295 | 0.0 | 0.194 |
| Anaerobic performance | Training frequency | >=3 sessions/week | 6 | 0.508 [0.052, 0.964] | 0.029 | 30.9 | 0.194 |
| Sport-specific performance | Training frequency | <=2 sessions/week | 4 | 0.453 [0.044, 0.862] | 0.030 | 0.0 | 0.931 |
| Sport-specific performance | Training frequency | >=3 sessions/week | 4 | 0.413 [-0.068, 0.894] | 0.092 | 17.3 | 0.931 |
| Aerobic capacity | Total training sessions | <10 sessions | 6 | 0.220 [-0.137, 0.576] | 0.227 | 0.0 | 0.545 |
| Aerobic capacity | Total training sessions | >=10 sessions | 5 | 0.374 [0.025, 0.722] | 0.036 | 0.0 | 0.545 |
| Anaerobic performance | Total training sessions | <10 sessions | 5 | 0.216 [-0.210, 0.642] | 0.321 | 0.0 | 0.481 |
| Anaerobic performance | Total training sessions | >=10 sessions | 6 | 0.429 [0.013, 0.845] | 0.043 | 40.7 | 0.481 |
| Sport-specific performance | Total training sessions | <10 sessions | 4 | 0.205 [-0.244, 0.653] | 0.371 | 0.0 | 0.169 |
| Sport-specific performance | Total training sessions | >=10 sessions | 4 | 0.626 [0.228, 1.025] | 0.002 | 0.0 | 0.169 |

Note. Positive Hedges' g values favor BFR combined with interval training. Subgroup p values refer to tests for subgroup differences.
